# Supplementary material for: Local Control Model of a Human Ventricular Myocyte: An Exploration of Frequency-Dependent Changes and Calcium Sparks
Source: Biomolecules. 2023 Aug 17;13(8):1259. doi: 10.3390/biom13081259 (PMC10452762; doi:10.3390/biom13081259)
Supplement: Supplementary file 1 [file biomolecules-13-01259-s001.zip › biomolecules-2514334-supplementary.pdf]

Local Control Model of Ca^{2+} Release of a Human Ventricular Myocyte Model: An Exploration to Frequency-Dependent Changes and Calcium Sparks

Jerome Anthony E. Alvarez, Mohsin S. Jafri, Aman Ullah

Supplementary Material

Majority of the equations, state transitions, and flux terms were developed from an existing spatiotemporal rat model by Tuan et al [1-3] and other contributions of calcium spark characteristics by Williams et al [4]. In addition, the 6-state LCC Markov model and the 3-state RyR model were adopted from the guinea-pig model by Paudel et al [5] stemmed from the aforementioned literatures. Further variation, development, and adjustments of the model into a physiological human cardiac myocyte is detailed in the original submitted text accompanied by this supplemental material.

1. Numerical Methods

The model is fully stochastic in terms of channel gating of RyRs and LCCs. The program was mainly written in Fortran and CUDA Fortran, using CUDA programming toolkit to run on Nvidia Fermi GPU, with some parts written in C++. The Euler method was used to solve the partial-differential equations (PDEs) of diffusion-reaction, and other ordinary differential equations (ODEs). The adaptive time-step ranging from 10 ns to 1 µs was used. When there is some activity, due to channel gating, the time-step will be reduced for numerical stability. The units in the systems are as follows: transmembrane potential - *mV*, membrane currents - $\mu A/cm^2$, the ionic concentration - μM (defined based on the corresponding volume), time - second (*s*). To handle the extremely large data generated by the 3D model, the HDF5 (Hierarchical Data Format) library is used [6], where the data exported in a compressed format that can be extracted later for further data analysis. The IDL language (Data Visualization Software, 2014) was used to perform all data visualization and data analysis. In this study of calcium waves, the two ions (K^+, Na^+) are kept constant for the duration of the simulation. Similarly, the corresponding currents are modeled as uniform too [7].

2. Model Equations and Parameters

2.1 Calcium release site (CRU)

Cardiomyocyte CRUs in cardiac dyad consist of couplons where L-type calcium channels (LCCs) in t-tubules are co-localized with ryanodine receptors (RyR2s; type-2 in cardiac cells) in junctional SR (JSR) membrane. The 20,000 CRUs in this model are composed of 9 individual LCCs and 49 RyRs. These CRUs are intricately connected through the complex organization of the network SR (NSR) which stores the main intracellular calcium in cardiac muscle cells. The differential equations describing Ca^{2+} at the release site are as follows:

$$\frac{d[Ca]_{ds}^{(i)}}{dt} = \frac{(J_{ryr}^{(i)} - J_{efflux}^{(i)} + (J_{dhpr}^{(i)})}{\lambda_{ds}} - 2\frac{d[CaM]^{(i)}}{dt} - \frac{d[CaSL]^{(i)}}{dt} - \frac{d[CaSR]^{(i)}}{dt}$$

$$\frac{d[CaM]^{(i)}}{dt} = k_{CaM}^{+}\left([Ca]_{ds}^{(i)}\right)^2\left([CM]_T - [CaM]_{ds}^{i}\right) - k_{CM}^{-}[CaM]_{ds}^{i}$$

$$\frac{d[CaSL]^{(i)}}{dt} = k_{SL}^{+}\left([Ca]_{ds}^{i}\right)\left([SL]_T - [CaSL]_{ds}^{i}\right) - k_{SL}^{-}[CaSL]_{ds}^{i}$$

$$\frac{d[CaSR]^{(i)}}{dt} = k_{SR}^{+}\left([Ca]_{ds}^{i}\right)\left([SR]_T - [CaSL]_{ds}^{i}\right) - k_{CM}^{-}[CaSR]_{ds}^{i}$$

where (i) is an index indicating the ith specific CRU, $\lambda_{ds} = V_{ds} / V_{myo}$ is the volume fraction that scale the fluxes, defined based on myoplasmic volume, to the subspace volume compartment. The membrane buffers used here are similar to those used previously [8, 9].

2.2 3-State Ryanodine Receptor Type-2 Model

To capture the Ca^{2+} release from CRUs, this model includes a 3-state ryanodine receptor mode switching (Figure 2) which incorporates cytosolic calcium-dependent and luminal calcium-dependent gating from Paudel and co-workers [5]. A luminal dependence function modulated RyR open probability to match calcium spark characteristics from Williams et al [10] and also incorporated minimal adjustments to allosteric coupling energies. The second closed state (C3) is the RyR2 adaptive state from changes in $[Ca^{2+}]_i$.

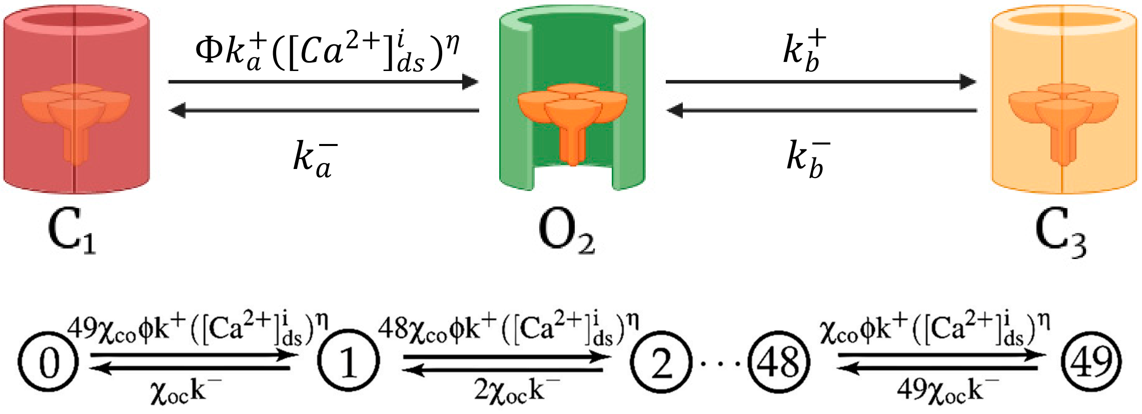

In this RyR2 model, the luminal regulation function (Φ) is determined by its dependency on luminal Ca^{2+} regulation coefficient Φ_m, and Φ_b

$$\Phi = \Phi_m [Ca^{2+}]_{sr} + \Phi_b$$

where $[Ca^{2+}]_{sr}$ represents both $[Ca^{2+}]_{jsr}$ and $[Ca^{2+}]_{nsr}$.

Luminal regulation function (Φ) modifies the channel opening rate, which eventually loads $[Ca^{2+}]_{sr}$ available for release. Coupling of the RyR2 channels in the cluster uses allosteric coupling energies, ε_{oo} and ε_{cc}, as described by Williams et al [10]. At each release site, the transition between two cluster states S_i and S_j is represented as:

$$S_i \underset{\chi_{OC}\, k_{ryr}^-\, N_{O\to C}}{\overset{\chi_{CO}\, k_{ryr}^+\, N_{C\to O}}{\rightleftarrows}} S_j$$

where $N_{O\to C}(N_{C\to O})$ represents the number of channels in cluster state S_i (S_j) that can be switched from Open to Closed (Closed to Open). The coupling gating in the cluster is given by the following equations:

$$\chi_{co} = \exp(-\bar{a}_j \times 0.5 \times (N_{closed}\mathcal{E}_{cc} - (N_{open} - 1)\mathcal{E}_{oo}))$$

$$\chi_{oc} = \exp\left(-\bar{a}_j \times 0.5 \times (N_{open}\mathcal{E}_{oo} - (N_{closed} - 1)\mathcal{E}_{cc})\right)$$

with $\overline{a_j}$ is the average allosteric connectivity for each RyR based on mean-field approach by Groff et al [11]. The variables N_{closed} and N_{open} represent the current number of non-conducting, and conducting channels in the cluster state Si, respectively.

Details of the 3-state RyR2 model are also detailed in section 2.2. and Appendix A.2 Supplementary Equations of the manuscript.

2.3 6-State L-Type Calcium Channel Model

The 6-state L-type Ca^{2+} channel (LCC) model is derived from 5-state LCC model for rat ventricle myocyte from Sun and co-workers with parameters adjusted for the new spark model [12]. Permeation of the L-type Ca^{2+} channel used the Goldman-Hodgkin-Katz (GHK) formalism was used [13].

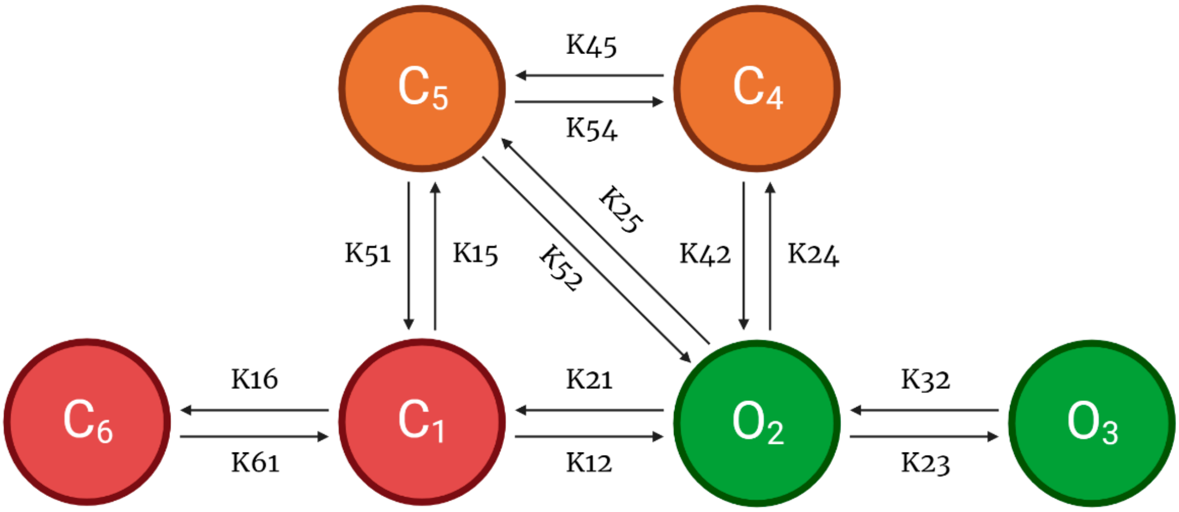

Even though the role of extracellular calcium was not investigated in this study, the model also considered the effect of extracellular calcium $[Ca^{2+}]_o$ on the model by modulating single channel current and shifting the half-activation voltage $V_{1/2}$:

$$V_{\frac{1}{2}} = -40.344 + 13.31 \times \exp(-[Ca]_o \times 10^{-3}/0.053) + 31.65 \times exp(-[Ca]_o \times 10^{-3}/11.305) + 3$$

It has been suggested that CaM tethers to a region of C-terminal between an EF and IQ domain of LCC [14], with 2 different segments (A and C sequences) within the pre-IQ motif of LCC have been indicated to be critical for CDI [15], and this tethering occurs at very low level of calcium concentration (20-100 nM) [16]. This is the range of resting calcium level; thus, it is assumed that all CaM tethers to the channels all the time. So, the total [CaM] is assumed fixed in the subspace, and calcium-dependent inactivation of LCC (k2→4) is modelled via calcium-Calmodulin (CaCaM) complex. In the model, similar to the experimental data, it is assumed that a single bound Ca^{2+}/CaM complex is both necessary and sufficient for CDI [17]. Hence, the transition rate for CDI is formulated as k24 = c24 [Ca^{2+}/CaM].

Not only Ca^{2+}, but also other ions can also permeate via LCC [18]. However, due to the large permeability of Ca^{2+} compared to other ions (e.g., PCa/PNa > 1000), in this study, only Ca^{2+} current is

modelled. Due to the nonlinearity in the I-V curve, and based on the assumption of independent permeation between ion species and constant-field theory, the GHK formalism was used [13].

Details of the 6-state LCC model are also explained in section 2.3 of the manuscript.

2.4 Na^+ Channel Current

The whole-cell Na^+ current was derived from the formula

$$I_{Na} = g_{Na} m_{Na} h_{Na}^3 j_{Na} (V_m - E_{Na})$$

with the ODE for gating variable follows the first-order differential equation [19]

$$\frac{dx_{Na}}{dt} = \frac{x_\infty - x_{Na}}{\tau_x}$$

where x represents the dimensionless gating variables, either m, h, or j, in the range between 0 and 1.0. The unit for the time constants is in seconds.

$$h_\infty = \frac{1}{1 + \exp\left((V_m + 76.1)/6.07\right)}$$

$$j_\infty = h_\infty$$

$$m_\infty = \frac{1}{1 + \exp\left((V_m + 48)/-6.5\right)}$$

$$\tau_m = \frac{0.00136}{0.32(V_m + 47.13)/(1 - \exp\left(-0.1(V_m + 47.13)\right) + 0.08 \times \exp\left(-V_m/11\right)}$$

If V_m >= -40

$$\tau_h = 0.0004537(1 + \exp\left((V_m + 10.66)/(-11.1)\right))$$

$$\tau_j = \frac{0.01163(1 + \exp\left(-0.1(V_{m+32})\right))}{\exp\left(-2.535 \times 10^{-7} V_m\right)}$$

If V_m < -40

$$\tau_h = \frac{0.00349}{0.81 \exp((V_m + 80)/(-6.8)) + 3.56 \exp(0.079 V_m) + 3.1 \times 10^5 \exp\left(0.35 V_m\right)}$$

$$\tau_j = \frac{0.00349}{\frac{5(V_m + 37.78)}{1 + \exp\left(0.311(V_m + 79.23)\right)}\left(-12714 \exp(0.2444V_m) - 3.474 \times 10^{-5} \exp(-0.04391V_m)\right) + \frac{0.1212\exp\left(-0.01052V_m\right)}{1 + \exp\left(-0.1378(V_m + 40.14)\right)}}$$

$$h_\infty = \frac{0.135\exp\left((V_m + 80)/(-6.8)\right)}{0.135 \exp\left(\frac{V_m + 80}{-6.8}\right) + 3.56 \exp(0.079V_m) + 3.1 \times 10^5 \exp\left(0.35V_m\right)}$$

$$j_\infty = \frac{\frac{V_m + 37.78}{1 + \exp\left(0.311(V_m + 79.23)\right)}\left(-12714 \exp(0.2444V_m) - 3.474 \times 10^{-5} \exp(-0.04391V_m)\right)}{\frac{V_m + 37.78}{1 + \exp\left(0.311(V_m + 79.23)\right)}\left(-12714 \exp(0.2444V_m) - 3.474 \times 10^{-5} \exp(-0.04391V_m)\right) + \frac{0.1212\exp\left(-0.010527V_m\right)}{1 + \exp\left(-0.1378(V_m + 40.14)\right)}}$$

2.5 K^+ Channel Currents

There are four different K^+ channel currents (I_{to}, I_{K1}, I_{Kr}, and I_{Ks}). The formulas for these currents are based on models developed using experimental data observed in humans [20-22]. Modified I_{to} is also detailed in section 2.4 of the manuscript. I_{Kr} and I_{Ks} formulations are adopted from O'Hara et al [23].

Inward Rectifier Potassium Current (I_{K1})

$$I_{K1} = g_{K1} \times \frac{[K^+]_o}{[K^+]_o + 210} \times \frac{(V_m - E_K - 1.73)}{1 + \exp\left(0.0896(V_m - E_K - 1.73)\right)}$$

Rapid Delayed Rectifier Potassium Current (I_{Kr})

$$I_{Kr} = g_{Kr} \times \sqrt{\frac{[K^+]_o}{5.4}} \times x_r \times R_{Kr} \times (V_m - E_K)$$

where

$$x_{r,\infty} = \frac{1}{1 + \exp\left(\frac{-(V_m + 8.337)}{6.789}\right)}$$

$$\tau_{xr,fast} = 12.98 + \frac{1}{0.3652 \cdot \exp\left(\frac{V_m + 31.66}{3.869}\right) + 4.123 \cdot 10^{-5} \cdot \exp\left(\frac{-(V_m - 47.8)}{20.38}\right)}$$

$$\tau_{xr,slow} = 1.865 + \frac{1}{0.06629 \cdot \exp\left(\frac{V_m + 34.70}{7.355}\right) + 1.128 \cdot 10^{-5} \cdot \exp\left(\frac{-(V_m - 29.74)}{25.94}\right)}$$

$$A_{xr,fast} = \frac{1}{1+\exp\left(\frac{V_m+54.81}{38.21}\right)}, \quad A_{xr,slow} = 1 - A_{xr,fast}$$

$$\frac{dx_{r,fast}}{dt} = \frac{x_{r,\infty} - x_{r,fast}}{\tau_{xr,fast}}$$

$$\frac{dx_{r,slow}}{dt} = \frac{x_{r,\infty} - x_{r,slow}}{\tau_{xr,slow}}$$

$$x_r = A_{xr,fast} \cdot x_{r,fast} + A_{xr,slow} \cdot x_{r,slow}$$

$$R_{Kr} = \frac{1}{\left(1 + \exp\left(\frac{V_m + 55}{75}\right)\right) + \left(1 + \exp\left(\frac{V_m - 10}{30}\right)\right)}$$

Slow Delayed Rectifier Potassium Current (I_{Ks})

$$I_{Ks} = g_{Ks} \times \left(1 + \frac{0.6}{1 + (\frac{3.8 \cdot 10^{-5}}{[Ca^{2+}]_i})^{1.4}}\right) \times x_{s1} \times x_{s2} \times (V_m - E_K)$$

where

$$x_{s1,\infty} = \frac{1}{1 + \exp\left(\frac{-(V_m + 11.60)}{8.932}\right)}$$

$$\tau_{x,s1} = 817.3 + \frac{1}{2.236 \cdot 10^{-4} \cdot \exp\left(\frac{V_m + 48.28}{17.80}\right) + 0.001292 \cdot \exp\left(\frac{-(V_m + 210)}{230}\right)}$$

$$\frac{dx_{s1}}{dt} = \frac{x_{s1,\infty} - x_{s1}}{\tau_{x,s1}}$$

$$x_{s2,\infty} = x_{s1,\infty}$$

$$\tau_{x,s2} = \frac{1}{0.01 \cdot \exp\left(\frac{V_m - 50}{20}\right) + 0.0193 \cdot \exp\left(\frac{-(V_m + 66.54)}{31}\right)}$$

$$\frac{dx_{s2}}{dt} = \frac{x_{s2,\infty} - x_{s2}}{\tau_{x,s2}}$$

It was reported that the transient outward current (I_{to}), present in human and rabbit atrial cells [24-27], has been shown to recover from inactivation at least two orders of magnitude faster in humans than in rabbits [27-29]. Thus, we also introduce a modification of I_{to} using O'Hara-Rudy activation and ten Tusscher-Panfilov inactivation gates. Furthermore, the modified I_{to} activation gate was decelerated (Equation 4). This is influenced by Courtemanche-Ramirez-Nattel (CRN) model where three activation gates are used (r^3), causing net activation to be slower and net deactivation to be faster than that of a single gate [29]. The ten Tusscher-Panfilov I_{to} epicardial inactivation time constant is also similar to CRN model [30], and this inactivation gate was further accelerated by adjusting to $s^{0.7}$ with the following equations:

$$I_{to} = G_{to} r^3 s^{0.7} (V_m - E_K)$$

$$r_\infty = \frac{1}{1 + exp\left(\frac{-(V_m - 14.34)}{14.82}\right)}$$

$$\tau_r = \frac{1.0515}{\frac{1}{1.2089 \times \left(1 + exp\left(\frac{-(V_m - 18.41)}{29.38}\right)\right)} + \frac{3.5}{1 + exp\left(\frac{V_m + 100}{29.38}\right)}}$$

$$s_\infty = \frac{1}{1 + exp\left(\frac{V_m + 20}{5}\right)}$$

$$\tau_s = 85 \times exp\left(\frac{-(V_m + 45)^2}{320}\right) + \frac{5}{1 + exp\left(\frac{V_m - 20}{5}\right)} + 3$$

where V_m is the membrane potential, r_∞ is activation gate with its time constant τ_r, s_∞ is inactivation gate with its time constant τ_s, and E_K is the reversal potential of K^+.

2.6 Sarcolemmal Pumps and Exchangers

The two extrusion pathways for calcium via SL are plasma-membrane Ca^{2+}/ATP-ase (PMCA) and Na^+/Ca^{2+} exchanger (NCX).

$$J_{ncx} = \frac{-A_m I_{ncx}}{F V_{myo}}$$

with A_m is the SL surface area and V_{myo} is the myoplasmic volume. The NCX is given by the formula

$$I_{ncx} = \bar{I}_{ncx} \frac{([Na]_i)^3 [Ca]_o \exp(\eta_{ncx} FV/(RT)) - ([Na]_o)^3 [Ca]_{myo} \exp((\eta_{ncx} - 1)FV/(RT)}{((K_{ncx,Na})^3 + ([Na]_o)^3)(K_{ncx,Na} + [Ca]_o)(1 + k_{ncx}^{sat} \exp((\eta_{ncx} - 1)FV/(RT)}$$

with $\bar{I}_{ncx}$ is the maximal NCX current. The formula for PMCA is

$$I_{pmca} = \bar{I}_{pmca} \frac{([Ca^{2+}]_{myo})^{\eta_{pmca}}}{(K_{m,pmca})^{\eta_{pmca}} + ([Ca^{2+}]_{myo})^{\eta_{pmca}}}$$

In the case of sodium and potassium pumps, we have

$$I_{Na^+/K^+} = \bar{I}_{Na^+/K^+} \frac{1}{(1 + 0.01245 \exp\left(-0.1\left(V_m \frac{F}{RT}\right)\right) \times 0.0365\left(\frac{1}{7}\exp\left(\frac{[Na]_o}{67300}\right) - 1\right)\exp\left(-V_m \frac{F}{RT}\right))} \times \frac{1}{1 + (\frac{21000}{[Na]_i})^{1.5} \frac{[K]_o}{[K]_o + 1500}}$$

2.7 Background Currents

The background currents follow the linear Ohm-law

$$I_{bX} = g_X(V_m - E_X)$$

The three different background currents were used in the model

$$I_{bCa} = g_{bCa}(V_m - E_{Ca})$$
$$I_{bNa} = g_{bNa}(V_m - E_{Na})$$
$$I_{bK} = g_{bK}(V_m - E_K)$$

with the reversal potentials are derived from Nernst equation.

$$E_X = \frac{RT}{Fz_X} \ln\left(\frac{[X]_i}{[X]_o}\right)$$

with z_x is the valance of ion X.

2.8 Sarco/Endoplasmic Reticulum Ca^{2+}-ATPase (SERCA)

The 2-state reduced model of the Tran-Crampin SERCA pump model [31] was chosen with parameters selected so that the maximum pumping rate per molecule, max(v_{serca}), is $5s^{-1}$.

$$J_{serca} = 2 \times Ap \times v_{serca}$$

$$v_{serca} = \frac{3.24873 \times 10^{12}(K_{myo})^2 + K_{myo}(9.17846 \times 10^6 - 11478.2K_{sr}) - 0.329904K_{sr}}{D_{cycle}}$$

$$D_{cycle} = 0.104217 + 17.923K_{sr} + K_{myo}(1.75583 \times 10^6 + 7.61673K_{sr}) + \left(K_{myo}\right)^2(6.08463 \times 10^{11} + 4.50544 \times 10^{11}K_{sr})$$

$$K_{myo} = \left(\frac{[Ca]_{myo}}{10^{-3}K_{d,myo}}\right)^2; \ K_{sr} = \left(\frac{[Ca]_{nsr}}{10^{-3}K_{d,sr}}\right)^2$$

2.9 Sarcoplasmic Reticulum Ion pumps

The three endogenous buffers of calmodulin (CaM), troponin (Trpn), and the phospholipids of the SR membrane (SRbuf) are used for the bulk myoplasm:

$$\frac{d[Ca;CaM]}{dt} = k_{CM}^{+}([Ca]_{myo}^{2})([CM]_T - [Ca;CaM]_{myo}) - k_{CM}^{-}[Ca;CaM]_{myo}$$

$$\frac{d[Ca;SRbuf]}{dt} = k_{SR}^{+}([Ca]_{myo})([SRbuf]_T - [Ca;SRbuf]_{myo}) - k_{SR}^{-}[Ca;SRbuf]_{myo}$$

$$\frac{d[Ca;Trpn]}{dt} = k_{Trpn}^{+}([Ca]_{myo})([Trpn]_T - [Ca;Trpn]_{myo}) - k_{Trpn}^{-}[Ca;Trpn]_{myo}$$

3. Parameters and Constants

3.1 Initial Ionic Concentrations

Parameter	Definition	Value
$[Na^+]_i$	Intracellular Na^+ concentration	1.4×10^4 µM
$[K^+]_i$	Intracellular K^+ concentration	1.4×10^5 µM
$[Ca^{2+}]_i$ or $[Ca^{2+}]_{myo}$	Intracellular/myoplasmic Ca^{2+} concentration	0.0854 µM
$[Ca^{2+}]_{nsr}$	NSR Ca^{2+} concentration	1.0×10^3 µM

3.2 Constants

Parameter	Definition	Value
F	Faraday Constant	9.6485×10^4 C mol^{-1}
R	Universal Gas Constant	8.314×10^3 mJ mol^{-1} K^{-1}
T	Temperature	310 Kelvin
$[Na^+]_o$	Extracellular Na^+ concentration	1.4×10^5 µM
$[Ca^{2+}]_o$	Extracellular Ca^{2+} concentration	1.8×10^3 µM
$[K^+]_o$	Extracellular K^+ concentration	5.4×10^3 µM

3.3 Cell Volume

Parameter	Definition	Value
V_{myo}	Myoplasmic volume	18 pL
V_{nsr}	NSR volume	0.86 pL
V_{jsr}	JSR volume	0.188 pL
V_{ds}	Dyadic Subspace volume	0.028 pL

3.4 Buffering Parameters (adopted from Sobie et al [32] and Grandi et al [21]).

Parameter	Definition	Value
β_{ds}	Dyadic subspace buffering fraction	0.1 (unitless)
β_{nsr}	NSR buffering	1.0 (unitless)
B_{myo}	Myoplasmic calmodulin buffer	1.4×10^2 µM
B_{jsr}	JSR calsequestrin buffer	3.0×10^4 µM
$K_{m,myo}$	Half-saturation constant for B_{myo}	0.96 µM
$K_{m,jsr}$	Half-saturation constant for B_{jsr}	6.3×10^2 µM
B_{trpn}	Total troponin concentration	70 µM
k_{on_trpn}	Troponin Ca^{2+} on rate	2.37 $\mu M^{-1}s^{-1}$
k_{off_trpn}	Troponin Ca^{2+} off rate	0.032 s^{-1}
$[B_{CaM}]_{Total}$	Total calmodulin buffer concentration	24 µM
k_{on_CaM}	Calmodulin Ca^{2+} on rate	100 $\mu M^{-1}s^{-1}$
k_{off_CaM}	Calmodulin Ca^{2+} off rate	38 s^{-1}
$[B_{SL}]_{Total}$	Total SL membrane buffer concentration	900 µM
k_{on_SL}	SL membrane Ca^{2+} on rate	115 $\mu M^{-1}s^{-1}$
k_{off_SL}	SL membrane Ca^{2+} off rate	1000 s^{-1}
$[B_{SR}]_{Total}$	Total SR membrane buffer concentration	47 µM
k_{on_SR}	SR membrane Ca^{2+} on rate	115 $\mu M^{-1}s^{-1}$
k_{off_SR}	SR membrane Ca^{2+} off rate	100 s^{-1}

3.5 SERCA Pump Parameters (adopted from Tran-Crampin model [31] with minimal adjustments).

Parameter	Definition	Value
A_p	Concentration of SERCA molecules	150 µM
K_{p_myo}	Dissociation constant (myoplasmic side)	169.8 µM
K_{p_nsr}	Dissociation constant (NSR side)	700 µM

3.6 Maximum Pump Densities

Parameter	Definition	Value
$\bar{I}_{PMCA}$	Plasmalemmal Ca^{2+} ATPase (PMCA) Pump	0.36 µA cm^{-2}
$\bar{I}_{NCX}$	Sodium-calcium exchange (NCX)	1.18×10^3 µA cm^{-2}
$\bar{I}_{NaK}$	Sodium-potassium ATPase (Na^+/K^+) Pump	1.8×10^3 µA cm^{-2}

3.7 Membrane and Ion Channel Conductances

Parameter	Definition	Value
g_{bCa}	Background Ca^{2+} conductance	9.1×10^{-5} mS cm^{-2}
g_{bK}	Background K^{+} conductance	3.2×10^{-3} mS cm^{-2}
g_{bNa}	Background Na^{+} conductance	5.97×10^{-4} mS cm^{-2}
g_{Na}	Fast Na^{+} channel conductance	16 mS cm^{-2}
g_{K1}	Inward rectifier K^{+} conductance	0.238 mS cm^{-2}
g_{Kr}	Rapid-delayed rectifier K^{+} conductance	0.0488 mS cm^{-2}
g_{Ks}	Slow-delayed rectifier K^{+} conductance	0.028 mS cm^{-2}
g_{to}	Transient outward K^{+} conductance	0.618 mS cm^{-2}
A_m	Cell membrane surface area	1.534×10^{-4} cm^{2}
C_{sc}	Specific membrane capacitance	1.0 μF cm^{-2}

3.8 RyR2 Parameters

Parameter	Definition	Value
$\bar{a}_j$	Average allosteric connectivity for each RyR2	3.571×10^{-2} (unitless)
ε_{cc}	Allosteric coupling energy at closed state	-1.022 (unitless)
ε_{oo}	Allosteric coupling energy at open state	-0.85 (unitless)
η_{RyR}	RyR2 cooperativity/Hill coefficient	2.2 (unitless)
Φ_m	Luminal Ca^{2+} regulation coefficient	2.4×10^{-4} (unitless)
Φ_b	Luminal Ca^{2+} regulation coefficient	2.0×10^{-2} (unitless)

3.9 L-type Ca^{2+} Channel Current Parameters

$$P_{o2} = \frac{-1}{1 + e^{(v - v_{1/2})/S}} + 1$$

$$V_{1/2} = -40.344 + 13.31 \times e^{\left(-\frac{[Ca]_o}{53.0}\right)} + 31.65 \times e^{\left(-\frac{[Ca]_o}{11305}\right)} + 3$$

$$S = (0.632 + 0.368 \times e^{(\frac{-[Ca]_0}{10^3} - 0.1)/0.784)}) \times (-3.792/(1 + e^{(\frac{[Ca]_0}{10^3} - 0.0469)/0.011)} + 6.39)$$

$$att = 130 + \frac{-129}{1 + e^{(V_m - 60)/8}} + \frac{0.76}{1 + e^{(V_m - 5)/3.14}}$$

$$\tau_{P_{O2}} = 0.00025 + 0.00305 \times e^{-0.0045(V_m + 7)^2} + 0.00105 \times e^{-0.002(V_m - 18)^2}$$

$$inch_{Ca} = 0.89 + \frac{-0.888}{1 + e^{(V_m - 43.066)/2.289}} \times (1.004 + \frac{-1.199}{1 + e^{([Ca]_o/10^3 - 0.259)/0.164}})$$

$$k_{1,5} = \frac{k_{5,1} k_{1,2} k_{2,5}}{k_{5,2} k_{2,1}}$$

$$k_{5,4} = \frac{k_{4,5} k_{5,2} k_{2,4}}{k_{4,2} k_{2,5}}$$

$$d_{dhpr} = d_{dhpr,([Ca]_0 = 1.8mM)} (1 + \frac{-1}{1 + e^{([Ca]_o/10^3 - 0.08)/0.0159}})$$

$$k_{1,2} = 0.115 \times P_{O2}/\tau_{O2}$$

$$k_{2,1} = 0.115 \times (1 - 0.155 \times P_{O2})/\tau_{O2} \times att$$

$$k_{2,3} = 0.7 \times (987.3 + \frac{-903.53}{1 + e^{(V_m - 51.23)/6.785}})$$

$$k_{3,2} = 500$$

$$k_{2,5} = 0.5 \times (32 + \frac{-18.46}{1 + e^{(V_m - 30)/5.746}}) \times (1 - inh_{Ca})$$

$$k_{5,2} = 1.0 \times \left(32 + \frac{-5.03}{1 + e^{(V_m - 30)/0.787}}\right) + (0.95 + \frac{-0.93}{1 + e^{(V_m + 40)/0.787}})$$

$$k_{2,4} = 8 \times [CaM]$$

$$k_{4,2} = 1$$

$$k_{5,1} = 6.9$$

$$k_{4,5} = 600$$

$$k_{3,2} = 500$$

$$k_{6,1} = 1 \times (0.0022 + \frac{2998.978}{1 + e^{-(Vm + 45) \times 0.71428}})$$

$$k_{1,6} = 1 \times (3000 + \frac{2998.978}{1 + e^{-(Vm + 45) \times 0.6667}})$$

4. Model Comparisons

Other well-known models in literature presented various characteristics suitable for different applications as discussed in the manuscript. In contrast, the model presented here also contain applicable properties as detailed in the supplementary table below:

Supplementary Table S1. Characteristic Comparisons to other Models

**Characteristic**	**Our Model**	**O'Hara-Rudy** (DOI: 10.1371/journal.pcbi.1002061)	**Tusscher-Panfilov** (DOI: 10.1152/ajpheart.00794.2003)	**Grandi-Bers** (DOI: 10.1016/j.yjmcc.2009.09.019)	**Himeno-Noma** (DOI: 10.1016/j.bpj.2015.06.017)
Action Potential	✓	✓	✓	✓	✓
Membrane Currents	✓	✓	✓	✓	✓
Formulation	Stochastic	Empirical	Empirical	Empirical	Stochastic
Calcium Sparks	✓	☒	☒	☒	☒
RyR Open Probability	✓	✓	☒	☒	✓
RyR Adaptability	✓	☒	☒	☒	☒
Calcium Cycling (SR-Myoplasm)	✓	✓	☒	✓	☒
Rate Dependence	✓	✓	✓	✓	✓
Dynamic Pacing (slow-fast-slow)	✓	☒	☒	☒	☒
AP Restitution (S1S2)	✓	✓	☒	☒	☒
RyR Transition States	3-state	☒	☒	☒	2-state
LCC Transition States	6-state	☒	☒	☒	4-state
CRU Dynamics	Markov	Hodgkin-Huxley	Hodgkin-Huxley	Hodgkin-Huxley	Markov

✓ - present

☒ - absent

References

1. Hoang-Trong, T.M., A. Ullah, and M.S. Jafri, *Calcium Sparks in the Heart: Dynamics and Regulation.* Res Rep Biol, 2015. **6**: p. 203-214.

2. Hoang-Trong, T.M., et al., *A Stochastic Spatiotemporal Model of Rat Ventricular Myocyte Calcium Dynamics Demonstrated Necessary Features for Calcium Wave Propagation.* Membranes, 2021. **11**(12): p. 989.

3. Hoang-Trong, M.T., et al., *Cardiac Alternans Occurs through the Synergy of Voltage- and Calcium-Dependent Mechanisms.* Membranes, 2021. **11**(10): p. 794.

4. Williams, George S.B., et al., *Dynamics of Calcium Sparks and Calcium Leak in the Heart.* Biophysical Journal, 2011. **101**(6): p. 1287-1296.

5. Paudel, R., *A Local-Control Model of the Guinea Pig Ventricular Myocyte Allows Understanding of Force-Interval Relations at the Calcium Spark Level.* Biophysical Journal, 2019. **116**(3): p. 113a.

6. HDF-group. *http://www.hdfgroup.org/HDF5/*. 2014; Available from: http://www.hdfgroup.org/HDF5/.

7. Yang, Z., et al., *Na+-Ca2+ exchange activity is localized in the T-tubules of rat ventricular myocytes.* Circ Res, 2002. **91**(4): p. 315-22.

8. Sobie, E.A., et al., *Termination of Cardiac Ca2+ Sparks: An Investigative Mathematical Model of Calcium-Induced Calcium Release.* Biophys J, 2002. **83**: p. 59-78.

9. Smith, G.D., et al., *A simple numerical model of calcium spark formation and detection in cardiac myocytes.* Biophys J, 1998. **75**: p. 15-32.

10. Williams, G.S., et al., *Dynamics of calcium sparks and calcium leak in the heart.* Biophys J, 2011. **101**(6): p. 1287-96.

11. Groff, J.R. and G.D. Smith, *Ryanodine receptor allosteric coupling and the dynamics of calcium sparks.* Biophys J, 2008. **95**: p. 135-54.

12. Sun, L., et al., *A model of the L-type Ca2+ channel in rat ventricular myocytes: ion selectivity and inactivation mechanisms.* J Physiol, 2000. **529 Pt 1**: p. 139-58.

13. Goldman, D.E., *Potential, Impedance, and rectification in membranes.* J Gen Physiol, 1943. **27**: p. 37-60.

14. Ehlers, M.D., G.J. Augustine, and R.O. Field, *Calmodulin at the channel gate.* Nature, 1999. **399**: p. 105-107.

15. Fallon, J.L., et al., *Crystal structure of dimeric cardiac L-type calcium channel regulatory domains bridged by Ca2+* calmodulins.* Proc Natl Acad Sci U S A, 2009. **106**(13): p. 5135-40.

16. Pitt, G.S., et al., *Molecular basis of calmodulin tethering and Ca2+-dependent inactivation of L-type Ca2+ channels.* J Biol Chem, 2001. **276**: p. 30794-802.

17. Mori, M.X., M.G. Erickson, and D.T. Yue, *Functional stoichiometry and local enrichment of calmodulin interacting with Ca2+ channels.* Science, 2004. **304**(5669): p. 432-5.

18. Hess, P., J.B. Lansman, and R.W. Tsien, *Calcium channel selectivity for divalent and monovalent cations. Voltage and concentration dependence of single channel current in ventricular heart cells.* J Gen Physiol, 1986. **88**: p. 293-319.

19. Hodgkin, A.L. and A.F. Huxley, *A quantiative description of membrane current and its application to conduction and excitation in nerve.* J Physiol, 1952. **117**: p. 25-71.

20. Bondarenko, V.E., et al., *Computer model of action potential of mouse ventricular myocytes.* Am J Physiol Heart Circ Physiol, 2004. **287**: p. H1378-403.

21. Grandi, E., F.S. Pasqualini, and D.M. Bers, *A novel computational model of the human ventricular action potential and Ca transient.* J Mol Cell Cardiol, 2010. **48**(1): p. 112-21.

22. ten Tusscher, K.H.W.J., et al., *A model for human ventricular tissue.* American Journal of Physiology-Heart and Circulatory Physiology, 2004. **286**(4): p. H1573-H1589.

23. O'Hara, T., et al., *Simulation of the Undiseased Human Cardiac Ventricular Action Potential: Model Formulation and Experimental Validation.* PLOS Computational Biology, 2011. **7**(5): p. e1002061.

24. Clark, R.B., W.R. Giles, and Y. Imaizumi, *Properties of the transient outward current in rabbit atrial cells.* The Journal of Physiology, 1988. **405**(1): p. 147-168.

25. Escande, D., et al., *Two types of transient outward currents in adult human atrial cells.* American Journal of Physiology-Heart and Circulatory Physiology, 1987. **252**(1): p. H142-H148.

26. Giles, W.R. and A.C. van Ginneken, *A transient outward current in isolated cells from the crista terminalis of rabbit heart.* The Journal of Physiology, 1985. **368**(1): p. 243-264.

27. Shibata, E.F., et al., *Contributions of a transient outward current to repolarization in human atrium.* American Journal of Physiology-Heart and Circulatory Physiology, 1989. **257**(6): p. H1773-H1781.

28. Fermini, B., et al., *Differences in rate dependence of transient outward current in rabbit and human atrium.* American Journal of Physiology-Heart and Circulatory Physiology, 1992. **263**(6): p. H1747-H1754.

29. Courtemanche, M., R.J. Ramirez, and S. Nattel, *Ionic mechanisms underlying human atrial action potential properties: insights from a mathematical model.* American Journal of Physiology-Heart and Circulatory Physiology, 1998. **275**(1): p. H301-H321.

30. Tusscher, K.H.W.J.t., et al., *A model for human ventricular tissue.* American Journal of Physiology-Heart and Circulatory Physiology, 2004. **286**(4): p. H1573-H1589.

31. Tran, K., et al., *A Thermodynamic Model of the Cardiac Sarcoplasmic/Endoplasmic Ca2+ (SERCA) Pump.* Biophysical Journal, 2009. **96**(5): p. 2029-2042.

32. Ramay, H.R., O.Z. Liu, and E.A. Sobie, *Recovery of cardiac calcium release is controlled by sarcoplasmic reticulum refilling and ryanodine receptor sensitivity.* Cardiovascular Research, 2011. **91**(4): p. 598-605.
